# Human Semen Samples with High Antioxidant Reservoir May Exhibit Lower Post-Cryopreservation Recovery of Sperm Motility

**DOI:** 10.3390/biom9030111

**Published:** 2019-03-19

**Authors:** Saleem A. Banihani, Razan F. Alawneh

**Affiliations:** Department of Medical Laboratory Sciences, Jordan University of Science and Technology, Irbid 22110, Jordan; razanalawneh98@yahoo.com

**Keywords:** human semen, cryopreservation, antioxidants, total antioxidant capacity, motility, sperm recovery

## Abstract

Cryopreservation-thawing of human semen was found to reduce the level of antioxidant activity surrounding the sperm, which may negatively affect post-cryopreservation (post-thaw) recovery of sperm motility. Therefore, the current manufactured cryoprotectant media have been supplemented with certain antioxidants to preserve the loss in seminal antioxidant activity. In this study, we aimed to explore the correlation between total antioxidant capacity (TAC) of human semen samples before cryopreservation and the post-thaw recovery of sperm motility. Normal semen specimens (*n* = 77) were recruited in this study. Sperm motility was measured for each semen sample before and after cryopreservation and the post-thaw recovery of sperm motility was calculated. Seminal TAC was measured spectrophotometrically before cryopreservation for each semen sample using the sensitive cupric ion-reducing antioxidant capacity (CUPRAC) method. The results from this study showed that the post-thaw recovery of sperm motility is negatively correlated (*p* = 0.0404, *p* = 0.0402) with the absorbance at 450 nm and the values of seminal TAC in terms of µM Trolox equivalents, as evaluated by CUPRAC, respectively. In conclusion, the total antioxidant reservoir in each ejaculated semen specimen could be a factor in determining the post-thaw recovery of sperm motility toward lower recovery for semen specimens of high antioxidant content.

## 1. Introduction

Semen cryopreservation is a procedure used in a variety of conditions that may affect semen quality such as surgical infertility intervention, chemotherapy treatment, seronegativity confirmation of viral infection (e.g., human immunodeficiency virus (HIV), hepatitis), and assisted reproductive technologies [1]. In spite of its practicality, however, cryopreservation significantly decreases sperm motility and viability. Therefore, it may not be practical in cases with poor semen quality (e.g., asthenozoospermia) [1]. Accordingly, various studies have targeted the improvement of this procedure by different means to ensure a better sperm recovery. One example of such a specific intention in research and technology was to augment the antioxidant system surrounding the sperm before cryopreservation, which may increase the post-cryopreservation (post-thaw) sperm recovery, and, hence, the ability of cryopreserved semen to achieve fertilization. This is, most of time, performed by enhancing/enriching the cryoprotectant medium of semen with some potential antioxidants [2,3,4].

In this work, we hypothesized that the total antioxidant capacity (TAC) in each individual semen sample is a factor in determining the post-thaw recovery of sperm motility. This suggestion is due to the fact that the amount of supplemented antioxidants in the manufactured cryoprotectant-medium is constant following a validated cryopreservation-thawing protocol. To test this hypothesis, we aimed to investigate the correlation between TAC of semen samples before cryopreservation and the post-thaw recovery of sperm motility.

## 2. Materials and Methods

### 2.1. Subjects and Sample Collection

Seventy-seven semen specimens with normal sperm parameters (sperm concentration > 15 × 10 ^6^ mL^−1^, total motility > 40%, total volume > 1.5 mL, [5]) were randomly collected over a 10-month period from different men (20–60 years old) who attended the andrology/in vitro fertilization unit at the King Abdullah University Hospital, Jordan. All samples were collected by masturbation after three days (~72 h) of sexual abstinence and were analyzed, according to the 2010 guidelines of the World Health Organization (WHO) [5].

After liquefaction, the semen sample was kept at 37 °C in the incubator until analysis. The sample volume was measured using a graded test tube with 0.1 ± 0.01 mL accuracy [5]. The sperm count was measured using a Makler chamber [5]. A preliminary evaluation of the sperm count was performed for each sample to determine the appropriate dilution [5,6]. Counting was undertaken using a light microscope (Olympus, Tokyo, Japan) at a magnification of 200×. Each sample was counted twice after homogenization by gentle vertexing and the assessment was performed by only one expert researcher to avoid the personal error and attain more accurate and precise measurements.

### 2.2. Ethical Considerations

The study was explained in detail to all recruited men by considering their education and culture. All subjects gave their informed consent for inclusion before they participated in the study. The study was conducted in accordance with the Declaration of Helsinki and the study protocol was approved by the Institutional Review Board (Code-20130097) of Jordan University of Science and Technology/King Abdullah University Hospital (Irbid-Al-Ramtha 22110, Jordan).

### 2.3. Experimental Design

Each semen sample was gently homogenized using a 1 mL pipette and analyzed for semen volume, sperm count, and sperm motility. Then the sample was divided into two aliquots. The first aliquot was tested for TAC and the second aliquot was cryopreserved in liquid nitrogen (−196 °C). After at least 72 h of cryopreservation, samples were thawed at room temperature and tested for sperm motility.

### 2.4. Assessment of Sperm Motility

A total amount of approximately 5 µL of each tested semen sample, after homogenization by gentle inversion, was used for assessment of motility using a micro-cell slide chamber (Conception Tech., San Diego, CA, USA) at 200× magnification [5,7]. Scanned fields were randomly selected and 200 spermatozoa per each replicate were assessed to achieve better accuracy in the measurements. The counting of motile sperm were performed quickly to avoid overestimation in the results.

### 2.5. Measurement of Total Antioxidant Capacity

Each sample was centrifuged at 300× *g* for 5 min and the supernatant (cell-free seminal plasma) was tested for TAC using the cupric ion-reducing antioxidant capacity (CUPRAC) method [8,9]. This method was adapted to measure the TAC of human semen because of its reliability, sensitivity, and suitability for biological fluids [9,10]. In addition, 1000 µL of working reagent (neocuproine alcoholic solution (0.0075 M), Cu (II) chloride (0.02 M), and ammonium acetate (NH_4_CH_3_CO_2_) buffer solution was mixed at 1:1:1 (*v/v/v*) with 12.5 μL of cell-free seminal plasma. All reaction tubes were centrifuged for 3 min at 750× *g*. The supernatant from each centrifuged sample was carefully collected. The absorbance of the product (colored complex) was measured against the reagent blank at 450 nm after 30 min incubation at room temperature. The absorbance at 450 nm was directly proportional with seminal TAC.

To express the values of seminal TAC, as evaluated by the CUPRAC method, we have used the standard antioxidant Trolox (6-hydroxy-2,5,7,8-tetramethylchroman-2-carboxylic acid) (Oxford Biomedical Research, Inc., Rochester Hills, MI, USA), which is a water-soluble analog of alpha-tocopherol. To do this, a Trolox calibration curve was generated from known Trolox concentrations and the data were expressed as µM Trolox equivalents.

### 2.6. Cryopreservation

In general, manufactured cryoprotectant media contain sterile purified water, glycerol, glucose, glycine, sodium citrate, egg yolk, and supported antioxidants.

An aliquot of the cryoprotectant medium (Irvine Scientific, Santa Ana, CA, USA) was added to the specimen and gently mixed for 5 min using a Hema-Tek aliquot mixer (Miles Scientific, Elkhart, IN, USA). This step was repeated to give a final 1:1 (*v/v*) ratio of the cryoprotectant medium to the semen sample. Cryovials containing the specimen were placed in the freezer at −20 °C for 8 min and in liquid nitrogen vapor at −80 °C for 2 h [7]. All vials were transferred to the liquid nitrogen at −196 °C. After at least 72 h, cryopreserved samples were thawed and analyzed for sperm recovery at 25 °C.

### 2.7. Statistical Analysis

Analyses of relationships between variables were performed using Spearman’s nonparametric correlation analysis, using GraphPad Prism 5.01 computer software (GraphPad Software Inc., San Diego, CA, USA). Differences were considered significant at *p* < 0.05.

## 3. Results

Table 1 demonstrates the mean values of the semen parameters (volume, count, and motility) for all semen specimens (*n* = 77) recruited in the study. All recruited samples had normal sperm parameters (sperm concentration > 15 × 10 ^6^ mL^−1^, total motility > 40%, total volume > 1.5 mL), according to the 2010 WHO guidelines [5].

**Figure 1:**
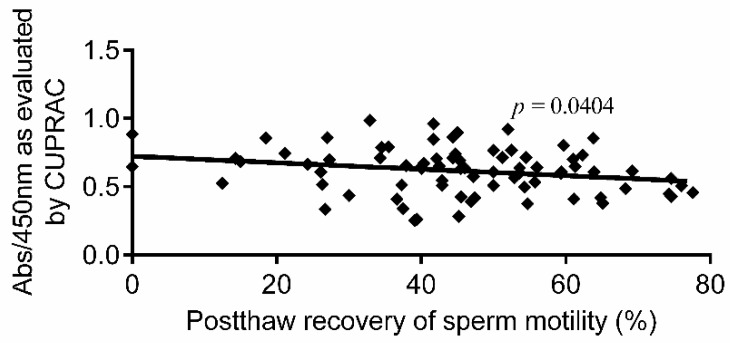
Correlation between post-thaw recovery of sperm motility and absorbance at 450 nm, as evaluated by the cupric ion-reducing antioxidant capacity (CUPRAC) method (*n* = 77). Abs: absorbance.

Figure 1 demonstrates the correlation between post-thaw recovery of sperm motility (*n* = 77) and the absorbance at 450 nm, as evaluated by the CUPRAC method. As illustrated in the figure, sperm recovery was negatively correlated (*p* = 0.0404, *r^2^* = 0.0680) with the absorbance at 450 nm, and, hence, with seminal TAC.

**Figure 2:**
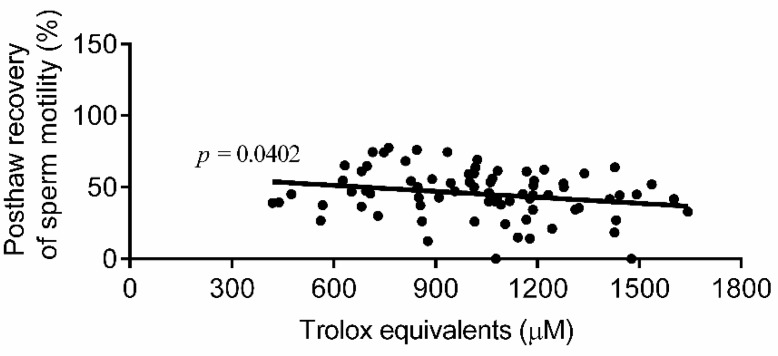
Correlation between post-thaw recovery of sperm motility and Trolox equivalents, as measured by the CUPRAC method (*n* = 77).

Figure 2 demonstrates the correlation between percentage of post-thaw recovery of sperm motility (*n* = 77) and the seminal TAC values in µM Trolox equivalents, as measured by the CUPRAC method at 450 nm. As shown in the figure, Trolox equivalents are negatively proportional (*p* = 0.0402, *r*^2^ = 0.0550) with the post-thaw recovery of sperm motility.

## 4. Discussion

In accordance with our hypothesis, the results from this work revealed that human semen samples with high antioxidant capacity may exhibit lower post-thaw recovery of sperm motility. These findings are in line with our central hypothesis.

Furthermore, the antioxidants are a double-edged sword. Adding antioxidants to human semen may induce adverse effects to sperm parameters, especially when used at high concentrations. For example, adding ascorbate (≥150 mM), vitamin E (≥20 mM), and l-carnitine (50 mM), a known antioxidants, to human semen in vitro significantly decreased progressive motility of sperm [11,12]. Moreover, human semen treatment with acetyl-l-carnitine did not improve sperm motility after cryopreservation-thawing [13]. Furthermore, ascorbate or carnosine (>50 mM) significantly reduced all post-thaw motility characteristics of ram sperm [14]. Furthermore, the in vitro study on human sperm by Donnelly et al. (1999) revealed that higher concentrations of ascorbate (>20 µM) induced adverse effects on sperm motility in both asthenozoospermic and normozoospermic semen samples [11].

In cellular systems, the imbalance between reactive oxygen species (ROS) [15], which are highly reactive oxidizing agents, and antioxidants to the favor of the former has been known as oxidative stress [16]. Studies have shown that the formation of oxidative stress state may lead to cell injury or cell death [17,18]. In human spermatozoa, oxidative stress and lipid peroxidation were found to be well-recognized factors behind the poor sperm function [19,20,21], and, hence, the reproductive outcomes [22]. *Vice versa*, it has been documented that high antioxidant levels in the cell may lead to a state termed as reductive stress, which may also induce adverse effects on cell function and cell viability [17]. It was documented that cells treated with high concentrations of antioxidants in vitro had higher levels of DNA oxidation when compared to the control [23]. The study by Menezo et al. (2007) revealed that patients treated with antioxidants for 90 days had unexpected adverse effects toward an increase in sperm decondensation (+22.8%) [24]. Therefore, in fact, oral antioxidants should not be recommended in males whose semen specimens exhibit a degree of de-condensation over a threshold of approximately 20% [24].

Vitamin C is the most abundant antioxidant in human semen [25]. Among antioxidants, vitamin C is considered as a powerful antioxidant since it has a strong reduction capability that can easily neutralize the generated free radicals, and, hence, reducing the oxidative damage. In this setting, its use is very beneficial as an antioxidant to protect the sperm, while it can have a prooxidant effects when used at high doses, especially in the presence of elevated amounts of transition metals such as ferric and cupric ions [26,27]. The seminal observations of pro-oxidant effects of vitamin C in vivo were confirmed in 1998 [27,28]. Earlier studies reported a possible pro-oxidant effects at higher amounts of vitamin C (500 mg day^−1^) [27,28]. Recent study conducted by Castro et al. (2017) revealed that ascorbic acid at a high dose (~5 mM) caused genotoxic as well as metabolic stresses in cultured glioma cells [29].

Unexpectedly, in the presence of transition metals (e.g., Cu^+2^, Fe^+3^), ascorbate can boost the formation of the harmful ROS. This pro-oxidant effect derives from the ability of ascorbate to reduce Cu^+2^ and Fe^+3^ to Cu^+2^ and Fe^+3^, respectively, which consequently reduces oxygen molecule (O_2_) to superoxide ion (O_2_•^_^) and stimulates the formation of hydroxyl radical (•OH) by Fenton’s reaction [30,31]. The damage to DNA, proteins, lipids, and other macromolecules in the cell results from the binding of either Cu^+2^ or Fe^+3^ to metal binding sites on these macromolecules, which is followed by the reaction of the metal complexes with hydrogen peroxide (H_2_O_2_) [32]. This leads to the production of ROS that may attack and damage the main functional groups in the cell [32].

Therefore, in this study, one possible reason behind the negative correlation between seminal TAC and the post-thaw recovery of sperm motility could be the content of the antioxidants in the cryoprotectant medium. Given that the antioxidant content is constant in the cryoprotectant medium (Absorbance/450 nm = 1.58 ± 0.06, *n* = 9) and varies between different semen samples, adding the cryoprotectant medium to the ejaculated semen samples of high antioxidant reservoir may upsurge the total antioxidants to a harmful level that may lead to the sperm injury.

In conclusion, human semen samples with high antioxidant capacity may yield lower post-thaw recovery of sperm motility. This may be because the final total antioxidant content surrounding the sperm after adding the cryoprotectant medium is not well optimized in each individual sample for semen ejaculates of a high antioxidant reservoir. However, these findings require further confirmation. While, as a whole, this work may open specific perspectives for further research gates to standardize/evaluate the final antioxidant content in the cryopreserved semen following the current cryopreservation protocols.

## Figures and Tables

**Table 1:** The mean values of the semen parameters for all specimens (*n* = 77). Values are means ± SEM (standard error of the mean).

Semen Parameter	Mean ± SEM
Total semen volume (mL)	3.44 ± 0.183
Sperm count (mL^−1^)	56.76 ± 5.08
Sperm motility before cryopreservation (%)	52.95 ± 2.07
Sperm motility after cryopreservation (%)	22.66 ± 1.51
Sperm recovery (%)	42.79 ± 1.82
